# Comparison of the effectiveness of Brandt-Daroff Vestibular training and Epley Canalith repositioning maneuver in benign Paroxysmal positional vertigo long term result: A randomized prospective clinical trial

**DOI:** 10.12669/pjms.343.14786

**Published:** 2018

**Authors:** Yaser Said Cetin, Omer Afsin Ozmen, Uygar Levent Demir, Fikret Kasapoglu, Oguz Basut, Hakan Coskun

**Affiliations:** 1 Yaser Said Cetin, Van Training and Research Hospital, Department of Otolaryngology, Van, Turkey; 2 Omer Afsin Ozmen, Uludag University Medical School, Department of Otolaryngology, Bursa, Turkey; 3 Uygar Levent Demir, Uludag University Medical School, Department of Otolaryngology, Bursa, Turkey; 4 Fikret Kasapoglu, Uludag University Medical School, Department of Otolaryngology, Bursa, Turkey; 5 Oguz Basut, Uludag University Medical School, Department of Otolaryngology, Bursa, Turkey; 6 Hakan Coskun, Uludag University Medical School, Department of Otolaryngology, Bursa, Turkey

**Keywords:** Benign positional paroxysmal vertigo, Brandt-Daroff exercises, Canalith repositioning maneuvers, Video electronystagmography

## Abstract

**Objective:**

Benign paroxysmal positional vertigo (BPPV) is the most common peripheral cause of vertigo. It can be defined as transient vertigo induced by rapid change in head position, associated with a characteristic paroxysmal positional nystagmus. Posterior canal benign paroxysmal positional vertigo is the most frequent form of BPPV. The aim of our study was to investigate and compare the effectiveness of Epley maneuver and Brand-Daroff home exercises for the treatment of posterior canal BPPV.

**Methods:**

A total of 50 patients between 27 and 76 years of age who were diagnosed with unilateral posterior canal into BPPV were randomized prospectively into two groups. In Group-1, modified particle repositioning maneuver and in Group-2 Brandt-Daroff exercises were utilized as treatment. Patients were followed weekly. Cure weeks were recorded. The patients were followed for 12 to 24 months for recurrence.

**Results:**

The recovery rates at the first, second and third week controls were 76%, 96% and 100% for Group-1 (modified Epley maneuver) and 64%, 88% and 100% for Group-2 (Brandt-Daroff exercises) respectively. The recovery rates were similar for both groups. The average follow-up duration was 18 months. In Group 1, 7 patients (28%) and in Group 2, 5 patients (20%) had recurrence, which was also similar with no statistical difference.

**Conclusion:**

It was concluded that both Brandt-Daroff Exercises and Modified Epley maneuvers were almost equally effective in the treatment of BPPV.

## INTRODUCTION

Benign paroxysmal positional vertigo (BPPV) is the most common cause of vertigo, with an incidence of 64/100,000. BPPV was first described by Barany in 1921, and a diagnostic maneuver was implemented in 1952 by Dix and Hallpike.^1^-^3^

The most common type of BPPV is posterior semicircular canal BPPV, with a rate of approximately 85%. Determination of the affected canal in BPPV is important for treatment. The most widely used tests for the diagnosis of BPPV and identifying the affected canal are the Dix-Hallpike test and Roll test. Several maneuvers based on cupulolithiasis and canalolithiasis theories have been proposed for BPPV treatment by Brandt, Daroff, Norre, Beckers, and McCabe.^4^–^6^ Presently, the most widely used maneuver for the treatment of posterior canal BPPV is the canalith repositioning procedure of Epley.^7^ In resistant cases and/or cases with suspicion of cupulolithiasis, Semont’s liberatory maneuver^8^ may be used. The main purpose of these maneuvers was to transport particles out of the canal towards the utricle with instant symptom resolution. On the other hand, vestibular physical therapy protocols such as Brandt-Daroff exercises are based on the principle of central compensation or the characteristic spontaneous resolution of BPPV and can be performed at home. The aim of this study was to compare the effectiveness of the Epley maneuver and Brandt-Daroff home exercises for the treatment of posterior canal BPPV and to observe differences in the resolution of BPPV on short- and long-term follow up.

## METHODS

This study was planned as a prospective, randomized, comparative study. Patients who applied to XXX University Medical School, Department of Otolaryngology, from March 2012 to May 2014 with complaints of vertigo and who were diagnosed with posterior canal BPPV by positional tests were included. The study was approved by the Ethics Committee of XXX University (03/01/2012 and 2012-1/3).

A detailed anamnesis of the patients regarding their symptoms was obtained (presentation of vertigo, predisposing factors, duration, recurrence of vertigo, accompanying hearing loss, tinnitus, sense of fullness, and sound intolerance). Patients were also questioned about neurologic symptoms (e.g., headache, facial paralysis, changes in mental status, slurred speech, loss of power, and syncope), visual problems (e.g., double vision, visual field constriction, and floating objects in front of the eye), and a history of systemic diseases, trauma, or drug usage. A physical examination was conducted that included otomicroscopy to examine the auricle, external ear canal, and tympanic membrane along with general otolaryngological and neurological examinations.

The diagnosis of BPPV was made on classical sudden, brief, recurrent rotatory vertigo attacks preceded by rapid and/or gravity-dependent head movements. All otological and neurological examination findings were normal.

**Fig.1:**
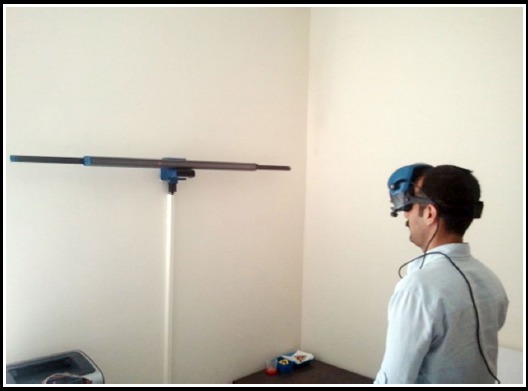
Patient undergoing a VENG test.

**Fig.2:**
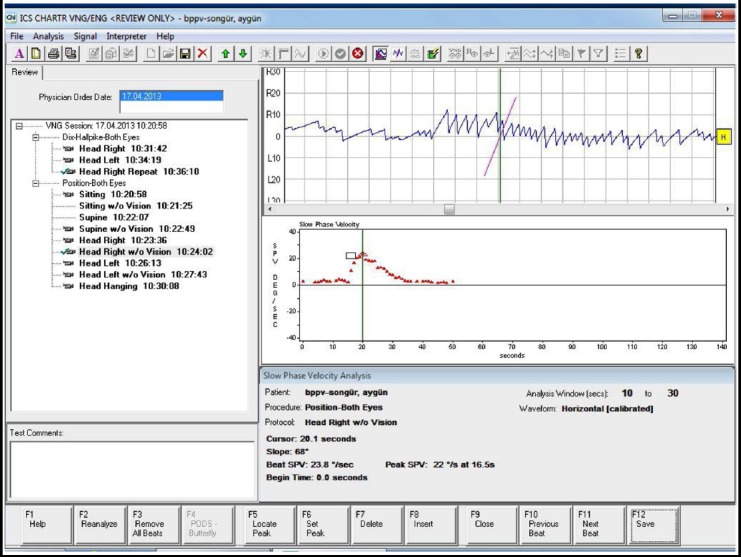
VENG test reports.

All patients with suspected BPPV underwent testing by videonystagmography (VNG) (ICS CHARTR 200; GN Otometrics A/S, Taastrup, Denmark) (Fig.1 and 2). Patients who underwent VNG were asked not to use any drugs that would affect the vestibular system for at least three days before the test, not to consume alcohol for up to 48 hours, to come with an empty stomach, and not to apply eye makeup. A VNG battery of gaze, saccade, tracking, optokinetic, static, and dynamic positional tests were performed followed by the Dix-Hallpike test. Numerical values for the saccadic, pursuit, optokinetic, and gaze tests were determined according to the values for the normal group. Any recorded nystagmus was evaluated in terms of normal distribution and angular velocity of the slow phase [slow phase velocity (SPV)]. During positional testing, nystagmus with an SPV average exceeding 5 degrees/second was considered an abnormal response. Patients considered having central vertigo or atypical forms of BPPV as a result of the anamnesis and physical examination was excluded from the study.

A total of 63 patients having complaints of positional vertigo were evaluated by VNG, and 50 patients having unilateral posterior canal BPPV were enrolled in the study. Two patients with anterior canal BPPV, eight patients with horizontal canal BPPV, two patients with bilateral posterior canal BPPV, and one patient with a diagnosis of central positional vertigo were excluded from the study.

For the diagnosis of BPPV, treatment maneuvers were performed with the help of VNG. The modified Epley maneuver was implemented in Group-1 as treatment. Brandt-Daroff exercises (five repeats three times a day) were explained orally and in writing to Group-2. Neither premedication nor bone vibrators were used during the maneuvers. Both groups were re-evaluated by VNG at the end of the first week. Patients without vertigo and nystagmus were considered healed; those having vertigo and/or nystagmus were considered treatment failures. Brandt-Daroff exercises and repositioning maneuvers were re-employed for patients who were not healed. All patients were monitored weekly until recovery.

The patients were followed for 12 to 24 months after recovery and re-evaluated for recurrence in case they had similar complaints. Neither a neck collar nor any kind of movement restriction was administered to the patients.

### Statistical analysis

A statistical analysis of the data was performed using SPSS Statistics Version.21 (IBM Corp., Armonk, NY, USA). The Kruskal-Wallis test was used to compare groups, and, in cases of statistical significance, a sub-group analysis was performed using the Mann-Whitney U test. Continuous variables showing a normal distribution are presented as the mean±standard deviation; otherwise, median (minimum - maximum) values are used. Categorical variables are given as (n) and (%). To compare categorical variables, Fisher’s exact chi-square test, Yates corrected chi-square test, and Fisher-Freeman-Halton tests were used; p<0.05 was accepted as the level of statistical significance. The statistical power was set at 0.63 in the analysis with a unit number of 25 in each of the groups in the study.

## RESULTS

Unilateral posterior canal BPPV was diagnosed in 50 patients (21 males and 29 females) between the ages of 27 and 76 (56.4±11.3) years. These patients were randomized into two groups by allocating successive patients to different groups. Twenty-five patients in Group-1 were treated with the modified Epley maneuver; patients in Group-2 were administered Brandt-Daroff home exercises for treatment. The groups were matched according to sex distribution (p=0.438) and patient age (p=0.470) or affected side (p=0.777) (Table-I).

**Table-I:** Demographic and clinical characteristics of the patients.

	Group-1 (Modified Epley)	Group-2 (Brandt-Daroff)
Age (year)	51.6±13.3	56.4±11.3
Sex (M/F)	10/15	11/14
Invovedside (right/left)	11/14	13/12
***Duration of complaints***		
≤1	20	19
1-6 months	4	3
>6 months	1	3
History of recurrent BPPV	9	7

An etiologic factor was identified in 20 patients (40%) (upper respiratory tract infection in 4 patients, vestibular neuritis in two patients, head trauma in three patients, long-term bed rest in two patients, otosclerosis surgery in four patients, bone anchored hearing aid implantation in one patient, and migraine in four patients), and 60% of the cases were accepted as idiopathic or primary BPPV.

The total duration of vestibular symptoms ranged between one day and two years. When categorized according to symptom duration, 39 patients had symptoms lasting less than one month, seven had symptoms between one and six months, and four had experienced symptoms for longer than six months. The groups were similar in terms of symptom duration (p=0.112).

The patients were questioned about their history of previous vertigo spells or BPPV diagnoses, and 16 patients were found to have recurrent BPPV. Nine of these patients were in Group-1 and seven were in Group-2 (p=0.532).

The outcomes were similar between Group-1 (modified Epley), where 19 patients (76%) were symptomless, and Group-2 (Brandt-Daroff), where 16 patients (64%) experienced recovery (p=0.537) in the first week. In the second week, recovery was achieved in five additional patients (20%) from Group-1 and six patients (21%) from Group-2; these numbers were similar (p=0.100). The remaining patients were recovered at the third week visit (one patient (4%) in Group-1 and three patients (12%) in Group-2) (p=0.609). Statistically significant differences between the recovery times were not detected between Groups 1 and 2 (p=0.537) (Table-II). Latency and the duration of nystagmus recorded using the Dix-Hallpike test were evaluated separately between groups (Table-III). Groups 1 and 2 were similar (p=0.132).

**Table-II:** The recovery weeks and rates of cases.

Recovery Week	Group-1 (Modified Epley)	Group-2 (Brandt-Daroff)
Week 1	19 (76%)	16 (64%)
Week 2	24 (96%)*	22 (88%)*
Week 3	25 (100%)*	25 (100%)*

* Cumulative numbers

**Table-III:** Nystagmus Start Time (Latency) and duration of nystagmus during Dix-Halpike test.

	Group-1 (Modified Epley)	Group-2 (Brandt-Daroff)
Latency*	9.1± 3.5	10.4±3.5
Duration*	23.5±14	18±9.5

* mean ± standard deviation

Based on VNG, nystagmus was detected during the gaze test in two patients who had a history of vestibular neuritis. In the remaining patients, VNG (gaze, saccades, optokinetic, and tracking tests) yielded normal results. Both of these patients belonged to Group-1 and their BPPV symptoms resolved with reposition maneuvers in the first week.

The patients were followed up for at least 12 months and up to 24 months. The average follow-up period was 18 months. In Group-1, seven patients (28%) experienced recurrence, compared to 5 patients (20%) in Group-2. When the two groups were evaluated in terms of recurrence, no significant difference was found (p=0.701).

## DISCUSSION

BPPV accounts for 20% of all hospital admissions with the complaint of vertigo, and the most common form of BPPV is posterior semicircular canal BPPV (relative frequency=90%). BPPV is usually a condition of the elderly; it is most commonly seen between the ages of 50 and 70 years. This is explained by age-related degenerative changes causing otoconial debris, which float freely and find their way into the semicircular canals, causing BPPV. The average age of the patients in our study was 56.4±11.3 years, consistent with previous reports. On the other hand, age is not a poor prognostic parameter for treatment success.^9^-^11^

Females are approximately 1.6–2 times more likely to experience BPPV than males. The female-to-male ratio of 1.6/1 in our study is in agreement with previous reports.^12^–^14^ In 50-60% of BPPV cases, the etiology cannot be determined and the condition is defined as primary or idiopathic. The causes of BPPV include inner ear diseases such as head trauma, vestibular neurinitis, labyrinthitis, and Meniere’s disease; middle ear diseases such as chronic otitis media, otosclerosis, or surgeries performed for these diseases; upper respiratory tract infections; prolonged surgery; and prolonged bed rest. Among these factors, the most common detectable etiologic factors are head trauma and vestibular neurinitis. In patients with a migraine, the BPPV incidence is reported to increase three fold.^15^,^16^ In our study, an etiologic factor was identified in 20 patients (40%); 60% of the cases were considered idiopathic or primary BPPV.

Spontaneous healing is common in the natural history of BPPV; however, to address the deterioration in quality of life and discomfort of patients, appropriate treatment for the underlying pathophysiology is required. Treatment options for BPPV include reposition maneuvers such as those of Epley and Semont, Brandt-Daroff habituation exercises, and medical and surgical treatments. The aim of these treatment strategies is to correct the symptoms in the shortest time and to prevent symptom recurrence. The preferred treatment methods are reposition maneuvers. For patients in whom reposition maneuvers are not possible, the preferred treatment method is Brandt-Daroff home exercises. Success rates between 80 and 100% have been reported for reposition maneuvers in BPPV.^17^ In a study by Cohen HS et al.^18^, 124 patients with posterior semicircular canal BPPV were divided into five treatment arms and the results were compared. Patients were evaluated in the first week using posturography scores and their long-term follow-up data were analyzed. It was found that the recovery rate was similar for the modified canalith repositioning maneuver, modified releasing maneuver, and Brandt-Daroff exercises, and these three options were superior to vestibular habituation exercises and the placebo maneuver. It has been proposed that Brandt-Daroff exercises could be used effectively instead of canalith repositioning maneuvers. Karanjai et al.^19^ randomized 48 patients to the Epley maneuver, Semont maneuver, or Brandt-Daroff exercises. Patients were evaluated during the second week and monitored for three months. The healing rate was 87% in the Epley group, 75% in the Semont group, and 56% in the Brandt-Daroff group. In another study conducted by Amor-Dorado JC et al.^20^, the efficacy of the Epley maneuver and Brandt-Daroff exercises for posterior semicircular canal BPPV treatment and recurrence rate after 48 months were evaluated. At the end of the first week, a recovery rate of 80.5% was achieved in the Epley group, whereas only 25% of patients in the Brandt-Daroff group were symptom-free. At the end of the first month, the recovery rates were 92.7% and 54.5% for Epley and Brandt-Daroff groups, respectively. The Epley maneuver was performed in nine patients who showed no healing in the Brandt-Daroff group at the end of the first week. It was concluded that the efficacy of the Epley maneuver was better during the early period. Patients were followed for 48 months and the recurrence rate was 36% in the Brandt-Daroff group and 35% in the Epley group, but the difference was not significant. Brandt and Daroff^5^ stated that after exercises were performed as primary treatment, 66 of 67 patients were healed within 14 days; however, two patients showed recurrence after a few months. They stated that they had identified a perilymph fistula in the remaining patient.^5^ The results of the Epley maneuver and Brandt-Daroff exercises were similar in our study. Both maneuvers achieved 100% recovery at the end of three weeks. The recurrence rate was 28% in the Epley group and 20% in the Brandt-Daroff group after 18 months of follow up.

Although previous studies have shown controversial results for Brandt-Daroff home exercises, they can be administered as primary treatment options for patients in whom the canalith repositioning maneuver cannot be performed (e.g., cervical problems or carotid stenosis). Additionally, Brandt-Daroff home exercises are applied to patients with BPPV, so patients can be treated at home alone. With failed recovery, patients may consult their physician. However, the diagnosis should be made correctly in order to not miss any other pathology. The use of VNG in the diagnosis of BPPV also increases the accuracy of the diagnosis;^21^ this is considered an advantage of the present study.

## CONCLUSIONS

Brandt-Daroff vestibular exercises are as effective as Epley canalith repositioning maneuvers in the treatment of BPPV with a similar low recurrence rate. Hence, both treatments may be utilized according to patient’s circumstances. However, the importance of making a correct diagnosis prior to treatment cannot be overestimated.

### Authors’ Contribution

**YSC** conceived, designed and did statistical analysis & editing of manuscript.

**OAO** drafting the article or revising it critically for important intellectual content.

**ULD, FK, OB** did data collection and manuscript writing.

**HC** takes the responsibility and is accountable for all aspects of the work in ensuring that questions related to the accuracy or integrity of any part of the work are appropriately investigated and resolved.
